# Gene Regulatory Network Inference from Pseudotime-Ordered scRNA-seq Data via Time-Lagged Divergence Measures

**DOI:** 10.1145/3774976.3774995

**Published:** 2025-12-22

**Authors:** Lingling Zhang, Tong Si, Lucas Koch, Haijun Gong

**Affiliations:** Department of Mathematics, State University of New York at Brockport, Brockport, NY, USA; Department of Health and Clinical Outcomes Research, Saint Louis University, St. Louis, MO, USA; Department of Mathematics and Statistics, Saint Louis University, St. Louis, MO, USA; Department of Mathematics and Statistics, Saint Louis University, St. Louis, MO, USA

**Keywords:** Applied Computing → Bioinformatics, Gene Regulatory Network, scRNA-seq Data, f-Divergence, Pseudotime Analysis, Integral Probability Metric, Partial Correlation

## Abstract

Inferring cell type-specific gene regulatory networks (GRNs) from time-series single-cell RNA sequencing (scRNA-seq) data is challenging due to sparse temporal resolution, high dimensionality, and inherent cellular heterogeneity. We present a novel integrative framework, called PseudoGRN, that unifies multiple pseudotime inference methods, different time-lagged divergence measures, non-redundant penalized network inference, and partial correlation analysis to reconstruct directed GRNs from time-series scRNA-seq data. Applying our method to the real-world scRNA-seq dataset, we demonstrate its superior performance over existing approaches, offering a robust and interpretable tool for uncovering dynamic regulatory mechanisms in single-cell systems.

## 1 Introduction

Single-cell RNA sequencing enables gene expression profiling at single-cell level, allowing researchers to identify distinct cell states, uncover differentiation trajectories, and investigate dynamic cellular processes [9, 13]. Analysis of scRNA-seq data has greatly deepened our understanding of complex biological processes and opened new avenues for the development of personalized medicine [27]. In particular, time-series scRNA-seq data further provides temporal information that can be used to reconstruct cell type-specific gene regulatory networks (GRNs) and investigate dynamic transcriptional regulation [5]. A variety of machine learning methods have been developed for GRN inference from omics data, including scRNA-seq, including Boolean networks [11], differential equation models [14], correlation networks [4], dynamic Bayesian networks [1, 18], regression-based frameworks [16, 28], and deep learning approaches [22, 24]. Most of these methods are based on canonical time, which reflects the actual chronological progression of molecular events. A major challenge in time-series scRNA-seq data analysis is the limited number of discrete time points, which reduces the temporal resolution and complicates the GRNs inference.

To address the challenges of network inference from time-series scRNA-seq data, particularly the sparse temporal sampling, and intrinsic cellular variability, pseudotime analysis [19] has emerged as a critical preprocessing step. Also known as trajectory inference, pseudotime methods estimate the relative progression of individual cells by ordering them along a continuous trajectory based on similarities in their gene expression profiles. This approach provides a high-resolution temporal framework that approximates dynamic biological processes without relying on explicitly measured time points. A variety of pseudotime inference methods have been developed to reconstruct cellular trajectories from single-cell transcriptomic data. These include graph-based approaches such as Monocle [26], Slingshot [23], PHATE [15], diffusion-based methods like Diffusion Pseudotime (DPT) [8] and Diffusion Maps [7], as well as probabilistic and velocity-based models such as scVelo [2].

Recently, Zeng et al.’s work [30] proposed a method called Normi, which integrates the Slingshot pseudotime algorithm with mutual information to infer gene regulatory networks. Normi has demonstrated promising performance, however, it also has several notable limitations. A major issue is the assumption that regulatory effects can have long time delays, suggesting that a gene’s expression may be influenced by distant past events, an idea often inconsistent with biological evidence. Most studies support the first-order Markov property [6], where the current state depends primarily on the immediate past. Thus, a time lag of one is typically sufficient and biologically more plausible for modeling regulatory dependencies [28]. Moreover, the estimation of the optimal time lag in Normi is computationally intensive, significantly limiting its scalability to larger datasets. Another limitation lies in its reliance on mutual information (MI) to infer regulatory relationships. Since MI is symmetric, it cannot capture directionality and is thus unsuitable for inferring causal interactions. Moreover, methods like Normi cannot distinguish between activation and inhibition, critical features of gene regulation, leading the inferred networks to resemble directed correlation networks rather than true regulatory networks.

To address these limitations, we propose a novel integrative framework, called PseudoGRN, that incorporates multiple pseudotime inference strategies, time-lagged f-divergence and integral probability metrics (IPMs), penalized non-redundant edge selection and partial correlation analysis within a unified platform for reconstructing directed gene regulatory networks from time-series scRNA-seq data. The remainder of this paper is organized as follows. Section 2 introduces the proposed methodology and algorithm. In Section 3, we evaluate the performance of our approach using real-world scRNA-seq datasets. Finally, Section 4 concludes the paper with a discussion of the results and directions for future research.

## 2 Methods

The time-series single-cell RNA sequencing dataset at any given time point t can be represented by a matrix $X^{\left(t\right)}\in R^{m\times n}$, where m denotes the number of genes and n denotes the number of cells. Our aim is to reconstruct the regulatory networks from the time-series scRNA-seq data. Since cells captured at the same experimental time point can be at different stages of biological progression, relying solely on discrete time points may obscure the true temporal dynamics. To more accurately estimate cellular progression, we apply different pseudotime inference methods to generate a continuous-valued vector representing the inferred temporal ordering of individual cells.

### 2.1 Pseudotime Inference

In this work, we implement several pseudotime inference methods, including Slingshot, PHATE, Diffusion Maps, PAGA, and PCA, to estimate cellular trajectories from scRNA-seq data. Below, we briefly describe two widely used approaches, Slingshot and PHATE. For details on Diffusion Maps and PAGA, readers are referred to the original publications [7, 29].

Slingshot [23] first constructs a minimum spanning tree (MST) over cell clusters to infer the global lineage structure, incorporating prior biological knowledge to guide the branching topology. Then, it fits simultaneous principal curves to model smooth, branching trajectories through the expression space. The pseudotime values are assigned to individual cells along each inferred lineage, providing a continuous and biologically meaningful temporal ordering that captures cellular progression.

PHATE [15] first computes local affinities between cells, models transitions using a diffusion process, and transforms the result into a heat potential representation to stabilize structure. Finally, non-metric multidimensional scaling embeds the data into a low-dimensional space, revealing smooth progression paths and branching trajectories. The pseudotime values are estimated by computing the Euclidean distances from each cell to a designated root cell along the PHATE manifold.

Since pseudotime inference can introduce noise and errors due to inaccuracies in temporal ordering, we adopt the approach of Zeng et al. [30], applying a sliding window (width k=5, step size 1) and average smoothing to construct representative, smoothed cell profiles along the pseudotime trajectory.

### 2.2 Time-lagged Divergence Measures

Previous studies [30, 31] have applied mutual information to infer gene regulatory networks (GRNs). In particular, the Normi method [30] infers regulatory links by computing time-delayed mutual information. However, due to the limitations discussed in the Introduction, we extend this idea and propose a more general framework that employs time-lagged f-divergence and integral probability metrics (IPMs) to quantify the influence of gene X on gene Y.

The time-lagged f-divergence quantifying the directional dependency between gene X at time t−l and gene Y at time t is defined as

$$D_{f}\left(P_{X}^{\left(t-l\right)}\|P_{Y}^{\left(t\right)}\right)=\int \;P_{Y}^{\left(t\right)}\left(z\right)f\left(\frac{P_{X}^{\left(t-l\right)}\left(z\right)}{P_{Y}^{\left(t\right)}\left(z\right)}\right)dz,$$

where, $P_{X}^{\left(t-l\right)}$ and $P_{Y}^{\left(t\right)}$ are the probability distribution functions of genes X and Y at time points t−l and t, respectively, with l denoting the time lag. The function f(u) is a proper, lower semi-continuous, and convex function satisfying f(1)=0. This formulation generalizes several well-known divergence measures, including Kull–back-Leibler, Jensen-Shannon, and Pearson divergence.

Similar to the time-lagged f-divergence, the time-lagged integral probability metric (IPM) is defined as:

$$D_{IPM}\left(P_{X}^{\left(t-l\right)}\|P_{Y}^{\left(t\right)}\right)=\underset{f\in ℱ}{sup}\;\left|E_{x∼P_{X}^{\left(t-l\right)}}[f(x)]-E_{y∼P_{Y}^{\left(t\right)}}[f(y)]\right|,$$

where ℱ is a class of bounded, measurable functions. The choice of ℱ determines the specific form of the divergence, such as the Wasserstein, Cramér and Energy distance.

In the previous study [30], the time lag was determined by maximizing the distance correlation between two genes, allowing the lag to be greater than one. This implies that gene expression could be influenced by events in a very distant past. However, biological studies support the Markov property in gene regulatory network modeling [6], which suggests that the current state of a gene is primarily influenced by its immediate past. Therefore, a time lag of one is typically sufficient and more biologically plausible for capturing regulatory dependencies [28]. Moreover, estimating the optimal lag in Normi [30] introduces substantial computational overhead. In our method, we adopt a first-order time lag assumption, l=1, to enhance both biological relevance and computational efficiency. A time-lagged divergence-based score between a transcription factor X and a target gene Z is defined as $D(X\|Z)=D\left(X_{t}\|Z_{t+1}\right)$, where D(⋅‖⋅) denotes either an f-divergence or an integral probability metric (IPM), which quantifies how changes in the expression of X at time t influence the expression of Z at a later time point t+1.

#### 2.3 Directed Network Inference Algorithm

Algorithm 1 outlines the pseudocode for the proposed divergence-based method PseudoGRN for inferring directed gene regulatory networks from scRNA-seq data. The procedure consists of four key steps: (1) pseudotime analysis, (2) computation of time-lagged divergence, (3) inference of non-redundant edges, and (4) identification of regulatory relationship.

**Algorithm 1:** Divergence-Based Directed Network Inference

**Require:** scRNA-seq data, Pseudotime methods, Divergence measures, Parameter λ.	
**Ensure:** Signed gene regulatory network	
1:	**Step 1: Pseudotime Analysis**
2:	Estimate pseudotime using trajectory inference methods (Slingshot, PHATE...)
3:	Sort the single cells according to pseudotime
4:	**Step 2: Computation of Time-lagged Divergence**
5:	**for all** gene pairs (X,Z) **do**
6:	Compute time-lagged divergence: $D(X\|Z)=D\left(X_{t}\|Z_{t+l}\right)$ using f-divergence or IPM.
7:	**end for**
8:	**Step 3: Inference of Non-redundant Edges**
9:	**for** each target gene Z **do**
10:	Initialize candidate TF set U (all TFs)
11:	Initialize selected TF set S←∅
12:	Rank all X∈U by descending D(X,Z)
13:	$X^{\left(1\right)}\leftarrow arg\;{max}_{X\in U}\;D(X,Z)$
14:	$S\leftarrow S\cup \{X^{\left(1\right)}\}$
15:	**while** S≠U **do**
16:	**for all** X∈U\S **do**
17:	Compute $D^{*}(X\|Z)=D(X\|Z)-\frac{\lambda }{\left|S\right|}\sum_{Y\in S}\;D(X\|Y)$
18:	**end for**
19:	$X^{*}\leftarrow arg\;{max}_{X\in U\backslash S}\;D^{*}(X\|Z)$
20:	$S\leftarrow S\cup \{X^{*}\}$
21:	**end while**
22:	**for all** X∈S **do**
23:	**if** $D^{*}(X\|Z)>0$ **then**
24:	Add edge X→Z to GRN
25:	**end if**
26:	**end for**
27:	**end for**
28:	**Step 4: Identification of Regulatory Relationship**
29:	**for all** inferred edges X→Z **do**
30:	Compute partial correlation $P_{XZ}$
31:	Label activation if $P_{XZ}>0$, inhibition if $P_{XZ}<0$
32:	**end for**

After computing the first-order time-lagged divergence-based scores D(⋅‖⋅) for all gene pairs using either f-divergence or IPM, next, we proposed a penalized variant of the max-relevance and min-redundancy (mRMR) strategy, originally introduced in [17] and later adapted for mutual information-based network inference in [30]. In our framework, we replace mutual information with divergence-based scores and incorporate a tunable penalization term, allowing for more flexible and accurate modeling of regulatory relationships. Step 3 in Algorithm 1 details this inference procedure. To infer regulatory links for a target gene Z, we first construct a candidate set of transcription factors U and initialize an empty selected set S. Genes in U are ranked in descending order based on their time-lagged divergence with Z, and the top-ranked gene is added to S. For each remaining candidate gene X∈U\S, we compute an adjusted divergence score which is defined as:

$$D^{*}(X\|Z)=D(X\|Z)-\frac{\lambda }{\left|S\right|}\sum_{Y\in S}D(X\|Y),$$

where the first term captures relevance to the target gene Z, and the second term penalizes redundancy with previously selected regulators, and λ is a turning parameter. This selection process is repeated iteratively to identify the most relevant and least redundant regulators, retaining only those with non-negative divergence scores as edges in the network.

The final step of Algorithm 1 determines the type of regulatory interaction, activation or inhibition. Following our recent work [28], we compute the partial correlation $P_{XZ}$ between each gene pair (X,Z) along the inferred edges from Step 3. A positive $P_{XZ}$ indicates activation, while a negative value suggests inhibition.

## 3 Results

In this section, we apply Algorithm 1 to reconstruct gene regulatory networks from time-series scRNA-seq data.

### 3.1 Data and Evaluation Metrics

To evaluate the performance of PseudoGRN, we analyze both simulated and real-world single-cell RNA-seq datasets. Due to space limitations, we present only the results on the real-world THP-1 dataset, which profiles the differentiation of THP-1 human myeloid leukemia cells into macrophages across eight time points, with 120 cells sampled at each time point [10]. The THP-1 dataset has been widely used as a benchmark for network inference [30].

To evaluate the performance of our method, we compute the Area Under the Receiver Operating Characteristic Curve (AUROC) and Area Under the Precision-Recall Curve (AUPRC), two standard metrics commonly used to assess the accuracy of predicted regulatory edges. The tuning parameter λ, which controls redundancy and sparsity of the inferred network in our algorithm, is selected via 5-fold cross-validation using Slingshot pseudotime and the Cramér divergence. The cross-validation results identify λ=1.5 as the optimal value, which is used in all subsequent analyses for GRN reconstruction.

### 3.2 Network Inference Analysis

We implement Algorithm 1 using five pseudotime inference methods, including Slingshot, PHATE, Diffusion Maps (DiffMap), PCA, and PAGA, and nine divergence measures: forward KL (F-KL), symmetric KL (S-KL), Jensen-Shannon (JS), Pearson, symmetric Pearson (S-Pearson), Neyman, Wasserstein (Wass), Energy, and Cramér divergence. In all experiments, each trial is repeated five times, and we report the average AUROC and AUPRC scores. Performance is assessed by comparing the inferred network against a known gene regulatory network composed of 20 genes [25].

Table 1 summarizes the mean AUROC and AUPRC scores for gene regulatory networks inferred using nine different divergence measures in combination with five pseudotime inference methods, all evaluated at the optimal regularization parameter λ=1.5. Standard errors are not reported, as their magnitudes are on the order of 10^−3^ and thus negligible. Our results indicate that, for a given divergence measure, the overall performance is relatively robust to the choice of pseudotime inference method. However, the performance is substantially affected by the choice of divergence measure, highlighting its critical role in network reconstruction.

**Figure 2:**
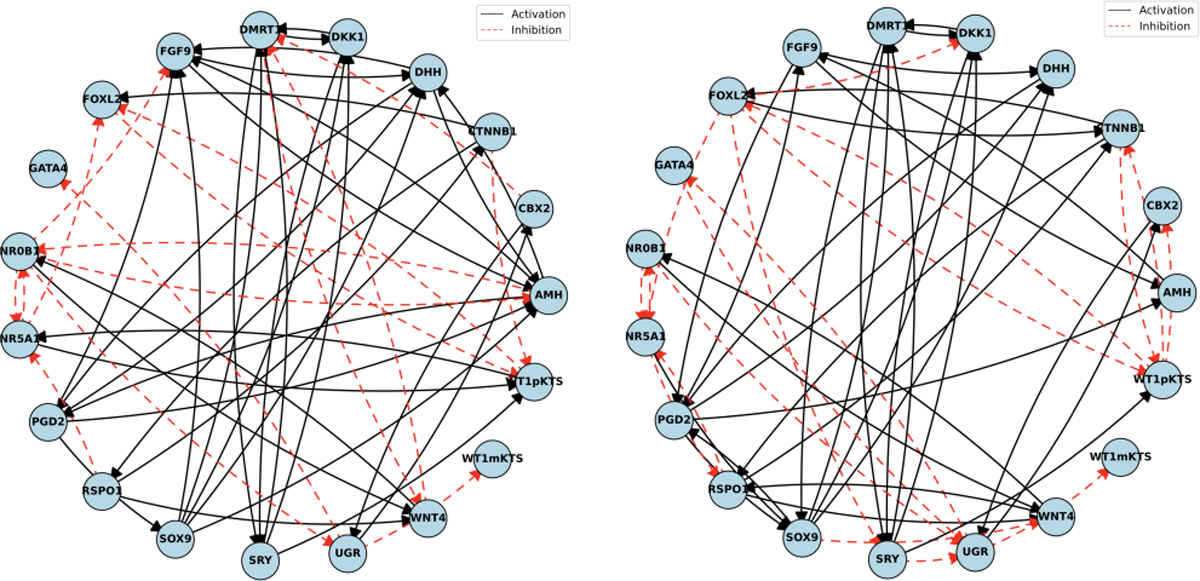
Gene regulatory networks inferred using Slingshot and Cramér with λ=1 (left) and λ=1.5 (right).

Among all the divergence measures, Cramér, a special case of the integral probability metric (IPM), consistently achieves the best performance, with a mean AUROC exceeding 0.6 and an AUPRC around 0.3 across nearly all pseudotime inference methods. This significantly outperforms baseline methods reported in [30], including DeepSEM, GRNBOOST2, SCODE, SCRIBE, GRNVBEM, LEAP, and SINCERITIES, whose mean AUROC scores typically hover around 0.5 and AUPRC values remain below 0.2. Under the Slingshot pseudotime, the AUROC of the Cramér-based method reaches approximately 0.65, while the computation time is only one-fifth of that required by Normi, which involves estimating an optimal time lag, a step that may not be biologically meaningful. Additionally, symmetric Pearson, Neyman, and standard Pearson divergences demonstrate competitive performance across various pseudotime strategies, further highlighting the robustness and effectiveness of divergence-based approaches. These results are consistent with Fig. 2, which presents boxplots of AUROC and AUPRC scores for each divergence measure, aggregated across five pseudotime inference methods. The boxplots illustrate the distribution of scores and their associated standard errors, highlighting the relative stability and performance of each measure.

To illustrate the inferred directed regulatory networks using our PseudoGRN method, Fig. 2 presents representative structures obtained using Slingshot and Cramér divergence under varying regularization parameters λ. The results show that both network structure and sparsity depend on λ; larger values of λ yield sparser networks with higher AUROC scores.

## 4 Conclusion

In this study, we introduce PseudoGRN, an integrative framework for inferring directed gene regulatory networks from scRNA-seq data. PseudoGRN incorporates several methodological innovations. First, multiple pseudotime inference methods are employed to estimate cellular trajectories. Next, we define a first-order time-lagged divergence-based score to quantify directional dependencies between gene pairs. A penalized, non-redundant edge selection strategy is then used to infer sparse, biologically meaningful network structures. Finally, partial correlation analysis is applied to classify each regulatory interaction as either activation or inhibition.

We evaluate the performance of our framework using real-world scRNA-seq data from THP-1 cells. Our experimental results demonstrate that the proposed approach outperforms all existing methods, including DeepSEM, GRNBOOST2, SCODE, SINCERITIES, and Normi. Notably, our results suggest that the overall performance is not highly sensitive to the choice of pseudotime analysis methods, but is significantly influenced by the choice of divergence function. Among the divergence measures evaluated, the Cramér distance achieved the best performance, substantially higher than those obtained by other divergence measures and all existing baseline methods. Compared to the Normi method, PseudoGRN demonstrates superior biological interpretability and computational efficiency.

Since scRNA-seq data often contain a large proportion of missing values that our current method cannot handle directly, one direction of future work is to incorporate our missing value imputation techniques [3, 12, 20] into this framework to enhance the accuracy of network inference. In addition, gene regulatory networks may exhibit non-stationary structures across different stages of cellular processes. Another future direction is to integrate changepoint detection algorithms [21] with pseudotime analysis to infer time-varying gene regulatory networks across different biological stages.

## Figures and Tables

**Figure 1:**
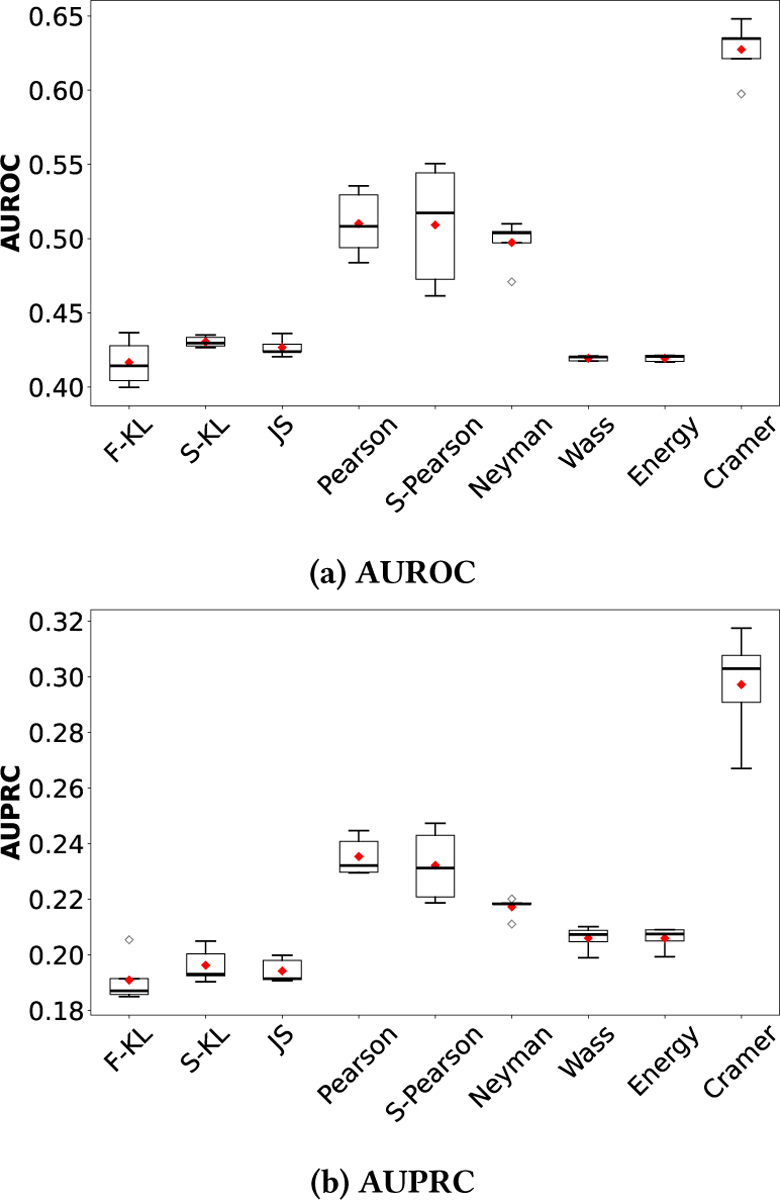
Boxplots of AUROC and AUPRC scores for each divergence measure, aggregated over five pseudotime methods.

**Table 1:** Mean AUROC and AUPRC scores using nine different divergence measures and five pseudotime inference methods.

Method	Slingshot	PHATE	DiffMap	PCA	PAGA

AUROC					

Cramér	0.6481	0.6213	0.6351	0.5975	0.6349
S-Pearson	0.5173	0.4614	0.5443	0.4727	0.5505
Neyman	0.5099	0.4972	0.5049	0.4709	0.5038
Pearson	0.5082	0.4838	0.5294	0.4939	0.5356
F-KL	0.4043	0.4366	0.4143	0.4278	0.3998
S-KL	0.4266	0.4351	0.4335	0.4277	0.4296
JS	0.4288	0.4361	0.4239	0.4204	0.4239
Wass	0.4209	0.4176	0.4205	0.4175	0.4202
Energy	0.4205	0.4168	0.4213	0.4172	0.4213

AUPRC					

Cramér	0.3175	0.2908	0.3030	0.2671	0.3078
S-Pearson	0.2312	0.2187	0.2430	0.2209	0.2474
Neyman	0.2201	0.2181	0.2186	0.2111	0.2183
Pearson	0.2321	0.2295	0.2408	0.2297	0.2447
F-KL	0.1857	0.2054	0.1871	0.1915	0.1849
S-KL	0.2050	0.2004	0.1931	0.1924	0.1903
JS	0.1980	0.1998	0.1907	0.1914	0.1910
Wass	0.1990	0.2048	0.2101	0.2073	0.2088
Energy	0.1993	0.2075	0.2090	0.2050	0.2090
